# Do physical fitness and cognitive function mediate the relationship between basic activities of daily living and quality of life in older adults with dementia?

**DOI:** 10.1007/s11136-023-03570-3

**Published:** 2023-12-19

**Authors:** Duarte Barros, Flávia Borges-Machado, Anabela Silva-Fernandes, Oscar Ribeiro, Joana Carvalho

**Affiliations:** 1 CIAFEL - Research Centre in Physical Activity, Health and Leisure, Faculty of Sport, University of Porto, Porto, Portugal; 2 ITR – Laboratory for Integrative and Translational Research in Population Health, Porto, Portugal; 3 CEGOT - Centre of Studies in Geography and Spatial Planning, Faculty of Arts and Humanities, University of Coimbra, Coimbra, Portugal; 4 Psychological Neuroscience Laboratory, School of Psychology, Psychology Research Center (CIPsi), University of Minho, Braga, Portugal; 5 CINTESIS@RISE - Center for Health Technology and Services Research at the Associate Laboratory RISE – Health Research Network, Aveiro, Portugal; 6 Department of Education and Psychology, University of Aveiro, Aveiro, Portugal

**Keywords:** Physical function, Cognition, Functionality, Major neurocognitive disorder

## Abstract

**Purpose:**

Independence in activities of daily living (ADLs) is associated with quality of life (QoL) in individuals with dementia. However, the contribution of physical and cognitive functions to this relationship needs further examination. This study aims to examine the mediating effect of physical fitness and cognitive function in the relationship between independence in basic ADLs and QoL among older adults with dementia.

**Methods:**

This cross-sectional study included 107 older adults with dementia (74.8% women; age 78.21 ± 7.70 years). Independence in basic ADL and QoL were evaluated using the Barthel Index (BI) and QoL- Alzheimer’s Disease Scale, respectively. The Alzheimer’s Disease Assessment Scale–Cognitive Subscale and the Mini-Mental State Examination were applied to assess cognitive function. Physical fitness was evaluated using the 30-s chair stand, 2-min step and the Timed-Up and Go tests. A structural equation modelling (SEM) with bootstrapping estimation was conducted to determine the relationship between all variables.

**Results:**

Independence in basic ADL positively affected QoL and this association was mediated by physical fitness (β = 0.242, p = 0.011). No statistically significant results were observed when testing cognitive function as a mediator between BI and QoL (β = 0.009, p = 0.345).

**Conclusions:**

Physical fitness (i.e., lower body strength, aerobic capacity, and mobility) plays a role in the relationship between basic ADL independence and QoL of older adults with dementia, reinforcing the need to improve and monitor these parameters throughout the disease progression. Future longitudinal studies should explore the temporal relationship between physical and cognitive function and its contribution to basic ADL independence and QoL.

## Introduction

Dementia is a major public health issue worldwide that results from diseases or injuries that affect the brain [1]. This syndrome is primarily characterized by a progressive deterioration in cognitive functions, in which its related symptoms are severe enough to interfere with independence in activities of daily living (ADLs) [2]. To date, the most recommended treatments are drug therapies and non-pharmacologic interventions that can temporarily slow the worsening of related symptoms, ameliorate functional capacity, and improve quality of life (QoL) [2].

QoL is a multidimensional construct that encompasses subjective and objective elements. Among its contributing factors, cognitive and functional abilities have often been highlighted [3–5]. Independence in ADLs is one of the cornerstones of every QoL framework in dementia, as functional impairment negatively impacts the QoL of people with dementia [6]. Complimentarily, the dysfunction in cognitive abilities is related to decreased QoL [7]; however, the available evidence is still not convincing as this relationship seems to be associated with behavioral and psychological symptoms such as depression [8, 9]. This inconsistency may also be attributed to the discrepancies in ratings of QoL domains between individuals with dementia and their carers [10] or to different QoL frameworks.

The ability to perform ADL is related to both physical fitness and cognitive function in individuals with dementia [11, 12]. Such association needs to be better established and likely requires a comprehensive understanding of the temporal changes in cognitive and physical function, as the deterioration in both often coincide [13]. While consistent evidence supports that cognitive impairment is a more robust predictor of physical decline than vice-versa [14, 15], it is also known that cognitive function deteriorates considerably faster succeeding physical disability [16, 17]. To date, few studies have demonstrated that frailty status, physical impairments and basic ADL disability may be noteworthy predictors of dementia onset [18–20]. Conversely, increasing evidence suggests that cognitive deterioration seems to precede and predict future functional decline in cases of mild dementia [21–23].

Cognitive decline plays a major role in the loss of autonomy and independence as there is a clear relationship between cognitive function and the ability to perform ADLs [24], mainly due to the progressive dysfunction of executive functions [25]. Indeed, as performance in instrumental ADLs is sensitive to cognitive decline, impairment in these ADLs tends to happen in the early stages of cognitive impairment [26] and is considered a clinical hallmark of dementia. In contrast, basic ADLs decline tends to occur in more advanced stages of dementia and may happen due to the combined effect of cognitive deterioration and (or be related to) motor changes and neurological manifestations [27].

Functional impairment and disability increase the care burden on caregivers and impact the QoL of individuals with dementia [28]. While physical performance is associated with QoL in individuals with dementia [29], the contribution of cognitive function to QoL perception seems erratic [30]. However, physical and cognitive functions are often examined separately, reinforcing the lack of information about their direct contribution to the QoL or their indirect effect on ADL performance. This study aims to examine the mediating effect of physical fitness and cognitive function in the relationship between independence in basic ADLs and QoL among older adults with dementia.

## Methods

### Study design and participants

This cross-sectional study was conducted in the city of Porto, Portugal. Participants were recruited from the project “Body and Brain”, which is registered at the US National Institutes of Health Clinical Trials registry—ClinicalTrials.gov (ID: NCT04095962); the study protocol can be found elsewhere [31].

The inclusion criteria were as follow: age ≥ 60, capable of walking independently without an assistive device or human assistance and clinically diagnosed with a major neurocognitive disorder or dementia using accepted diagnostic criteria such as those established by the Diagnostic and Statistical Manual of Mental Disorders (DSM-IV-TR or DSM-5), International Classification of Diseases 10th Revision (ICD-10), or the National Institute of Neurological and Communicative Diseases and Stroke/Alzheimer's Disease and Related Disorders Association (NINCDS-ADRDA) [32, 33]. Individuals were excluded from this study if data was missing on QoL-Alzheimer’s Disease Scale (QoL-AD) [34, 35].

Participants and their caregivers or proxy decision-makers received a complete explanation of the study procedures and signed the informed consent in full compliance with the Helsinki Declaration.

Data were collected between October 2018 and October 2019 in two different appointments. At the first appointment, sociodemographic and clinical data were gathered, and all physical domain tests were performed. Cognitive domains and self-rated QoL were evaluated on the second appointment in the following days. Questionnaires referring to people with dementia daily functionality and proxy-rated QoL were obtained from caregivers via scheduled in-person interviews afterward.

This study followed the Strengthening the Reporting of Observational Studies in Epidemiology (STROBE) Statement checklist for cross-sectional studies.

This study was approved by the Ethical Committee of the Faculty of Sports of the University of Porto (Ref CEFADE22.2018).

## Measures

Participants’ sociodemographic (e.g., age, years of education) and clinical information (e.g., dementia subtype, pharmacological treatment) were gathered via a structured questionnaire.

### QoL perception

The Portuguese version of QoL-AD was applied [34]. This instrument includes both reports from patients and caregivers on how the person with dementia feels regarding 13 domains: physical health, energy, mood, living situation, memory, relationship with family members, marriage and friends, ability to do things for fun, ability to do usual activities, and financial situation. The total score ranges from 13 to 52, with higher scores indicating better QoL [35].

### ADL independence

Participants’ ability to function independently in ADL was assessed via caregiver/proxy report using the Portuguese version of Barthel Index (BI) [36]. This instrument addresses ten basic daily activities (feeding, bathing, grooming, dressing, using the toilet, bowels, bladder, transfer, mobility, and stairs) with a total score ranging from zero to 100. Lower scores indicate higher dependency levels [37].

### Physical fitness

Lower body strength was assessed using the 30 s chair stand test from Senior Fitness Test (SFT) battery [38]. Participants completed as many stands as possible within 30 s; the score was the total number of stands performed properly during such timeframe [39].

Aerobic endurance was assessed using the 2-min steps test from the SFT battery [38]. Participants were asked to step in place as fast as possible for two minutes while raising their knees to a height halfway between the iliac crest and the middle of the patella. The final score was defined as the number of right-side steps of the criterion height [40].

The Timed-Up and Go test (TUG) [41] is a gold-standard test to evaluate older adults’ functional mobility that assesses agility and balance. Participants were requested to rise from a standard armchair, walk at a normal pace at a 3-m distance, turn around the ground mark positioned in front, return, and sit down again. The lowest time of the two trials was considered [41].

### Cognitive function

The Portuguese version of the Alzheimer’s Disease Assessment Scale–Cognitive Subscale (ADAS-Cog) was used to assess cognitive function [42]. This instrument examines features of cognitive decline such as memory, praxis, constructive ability, language, and orientation. Scores range from 0 to 68, with higher scores suggesting greater severity of cognitive impairment. Also, the Portuguese version of the Mini-Mental State Examination (MMSE) was applied [43]. This 30-point questionnaire is organized into six cognitive domains: orientation, retention, attention and calculation, delayed recall, language, and visuo-constructive ability. Lower scores suggest a higher cognitive impairment.

### Statistical analyses

Descriptive characteristics of the sample were presented as mean and standard deviation (SD) or median and interquartile range [IQR] for continuous variables and as frequency and percentages for categorical variables (Table 1). Missing values for BI (N = 1), 30 s chair stand (N = 2), and 2-min step (N = 2) were treated as a series of the mean using the mean value substitution method. All variables were evaluated for normality of distribution using a combination of histograms and the Kolmogorov–Smirnov test. Six of the seven variables included in the model were normally distributed (p > 0.05). The BI variable was not normally distributed. The time spent on the TUG test and ADAS-Cog score were inverted to have the same direction as the other latent physical fitness and cognitive function variables.

**Table 1:** Sample characteristics

Characteristics	Total (*n* = 107)
Age, years	78.21 (7.70)
Age range, years	61–94
Female, No. (%)	80 (74.8%)
Civil Status, No. (%)	
Single	1 (0.9%)
Widow	45 (42.1%)
Married or Civil Union	50 (46.7%)
Divorced or separated	11 (10.3%)
Dementia Subtypes, No. (%)	
Alzheimer’s disease	40 (37.4%)
Unspecified	37 (34.7%)
Vascular	10 (9.3%)
Multiple etiologies	10 (9.3%)
Other subtypes	10 (9.3%)
Formal education, years	4.00 [4.00–9]
Daily medications, No	7.00 [5.00–9.00]
Comorbidities, No	3 [2–4]
30-s chair stand test, Reps	9.61 (3.61)
2-min step test, Reps	48.57 (18.06)
TUG, s	13.15 [10.62 – 17.72]
ADAS-Cog, score	35.61 (13.58)
MMSE, score	17.84 (5.93)
QoL-AD, score	30.83 (4.56)
BI, score	90 [80–100]

*ADAS-Cog* Alzheimer’s disease assessment scale–cognitive subscale, *BI* Barthel index, *MMSE* mini mental state examination; *No* number; *QoL-AD* quality of life-Alzheimer’s disease scale, *Reps* repetitions, *S* seconds, *TUG* timed-up-and-go

Partial Pearson correlations (with bootstrap corrections, 5000 iterations and 95% confidence interval), controlled for age and gender were computed. Cronbach’s alpha determined the internal consistency of tests included in the questionnaire. Cronbach’s α was interpreted as follows: ≥ 0.07 as acceptable, ≥ 0.80 as good and ≥ 0.90 as excellent [44].

A Structural Equation Modeling (SEM) analysis was performed including BI as an exogenous variable; and physical fitness (TUG, 2 min-step and, 30 s chair stand), cognitive function (ADAS-cog and MMSE), and QoL-AD as endogenous variables. SEM was used to examine the mediating effect of cognitive function and physical fitness in the relationship between physical independence in ADLs and QoL. SEM refers to a statistical technique that combines an exploratory factor analysis and multiple regression, allowing for dealing with numerous variables, and testing hypotheses about how constructs are theoretically linked and the directionality of significant relationships. This method also provides an evaluation of how an “M” variable can mediate the relationship between two “X and Y” variables [45–47]. The fit of the models was calculated based on the following multiple criteria: X^2^ test, goodness-of-fit index (GFI) ≥ 0.95, comparative-fit index (CFI) ≥ 0.90, normed fit index (NFI) ≥ 0.95, standardized root mean square residual (SRMR) < 0.08, and root mean square error of approximation (RMSEA) < 0.08 [48]. Hypotheses regarding the structural relationships of the constructs explored in the model were evaluated using the magnitude of path coefficients, standardized coefficient, and their significance. Bootstrap corrections with 500 iterations and a 95% confidence interval were applied to the indirect effects [49].

All analyses were performed using SPSS 28 (SPSS, Inc., Chicago, IL, USA) and AMOS 28.0. A p-value of < 0.05 was considered statistically significant.

## Results

A total of 112 older adults were eligible for this study. Of those, five were excluded due to missing data in QoL-AD. The final sample comprised 107 older adults with ages ranging between 61 and 94 years (74.8% women; mean age 78.21 ± 7.70 years). The most common dementia subtypes were due to Alzheimer’s disease (37.4%) or unspecified causes (34.6%); most situations (69.1%) were diagnosed for a median of 3 [1–4] years. Sample characteristics are presented in Table 1.

Partial correlations controlling for age and gender are presented in Table 2. Overall, ADL was positively correlated with QoL (r = 0.310, p = 0.001), lower body strength (r = 0.479, p < 0.001), aerobic endurance (r = 0.495, p < 0.001) and functional mobility (r = 0.541, p < 0.001), while being negatively correlated with cognitive function assessed by ADAS-Cog (r = − 0.203, p = 0.038).

**Table 2:** Partial correlations controlling for age and gender

Variables	1	2	3	4	5	6	7
1. BI	**–**						
2. QoL	0.310*	**–**					
3. 30-s chair stand	0.479**	0.349**	**–**				
4. 2 min-step	0.495**	0.358**	0.625**	**–**			
5. TUG	0.541**	0.367**	0.637**	0.658**	**–**		
6. ADAS-Cog	− 0.203*	− 0.068	− 0.124	− 0.187	− 0.124	**–**	
7. MMSE	0.182	0.188	0.241*	0.093	− 0.143	0.826**	**–**

*ADAS-Cog* Alzheimer’s disease assessment scale–cognitive subscale, *BI* Barthel index; *MMSE* mini mental state examination, *QoL-AD* quality of life-Alzheimer’s disease scale, *TUG* timed-up-and-go

*p < 0.05; **p < 0.001

All measures presented an acceptable to excellent internal consistency with the exception of MMSE (α = 0.686; Table 3).

**Table 3:** Cronbach’s alpha values for the variables included in the model

Latent variables	Observed variables	No of items	Mean (SD)	Cronbach’s α
1. BI	Barthel index	10	86.6 (14.84)	0.805
2. Cognitive function	ADAS-COG	11	35.65 (13.64)	0.921
3. Cognitive function	MMSE	6	17.86 (5.93)	0.689
4. QoL-AD patient	QoL	13	31.31 (5.62)	0.818
5. QoL-AD caregiver	QoL	13	29.89 (5.21)	0.773

*ADAS-Cog* Alzheimer’s disease assessment scale–cognitive subscale, *BI* Barthel index, *MMSE* mini mental state examination, *QoL-AD* quality of life—Alzheimer’s disease scale

**Fig. 1:**
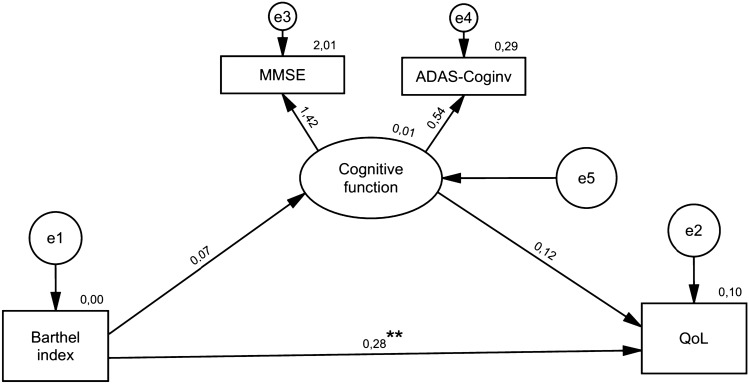
The mediating effect of cognitive function in the relationship between basic ADL and QoL

Two models were constructed to examine the mediating effect of cognitive function and physical fitness in the relationship between physical independence in ADLs and QoL. We did not find statistically significant results when testing cognitive function as a mediator between BI and QoL (β = 0.009, p = 0.345). Namely, BI did not exert a significant direct effect on cognitive function (β = 0.072, p = 0.361) nor on QoL (β = 0.118, p = 0.297). Additionally, BI directly affected QoL (β = 0.277, p = 0.002) (Fig. 1).

The fit of the model was met according to the following parameters: X^2^(1) = 0.303, p = 0.582, SRMR = 0.0162, RMSEA < 0.001, GFI = 0.999, NFI = 0.997 and CFI = 1.000.

**Fig. 2:**
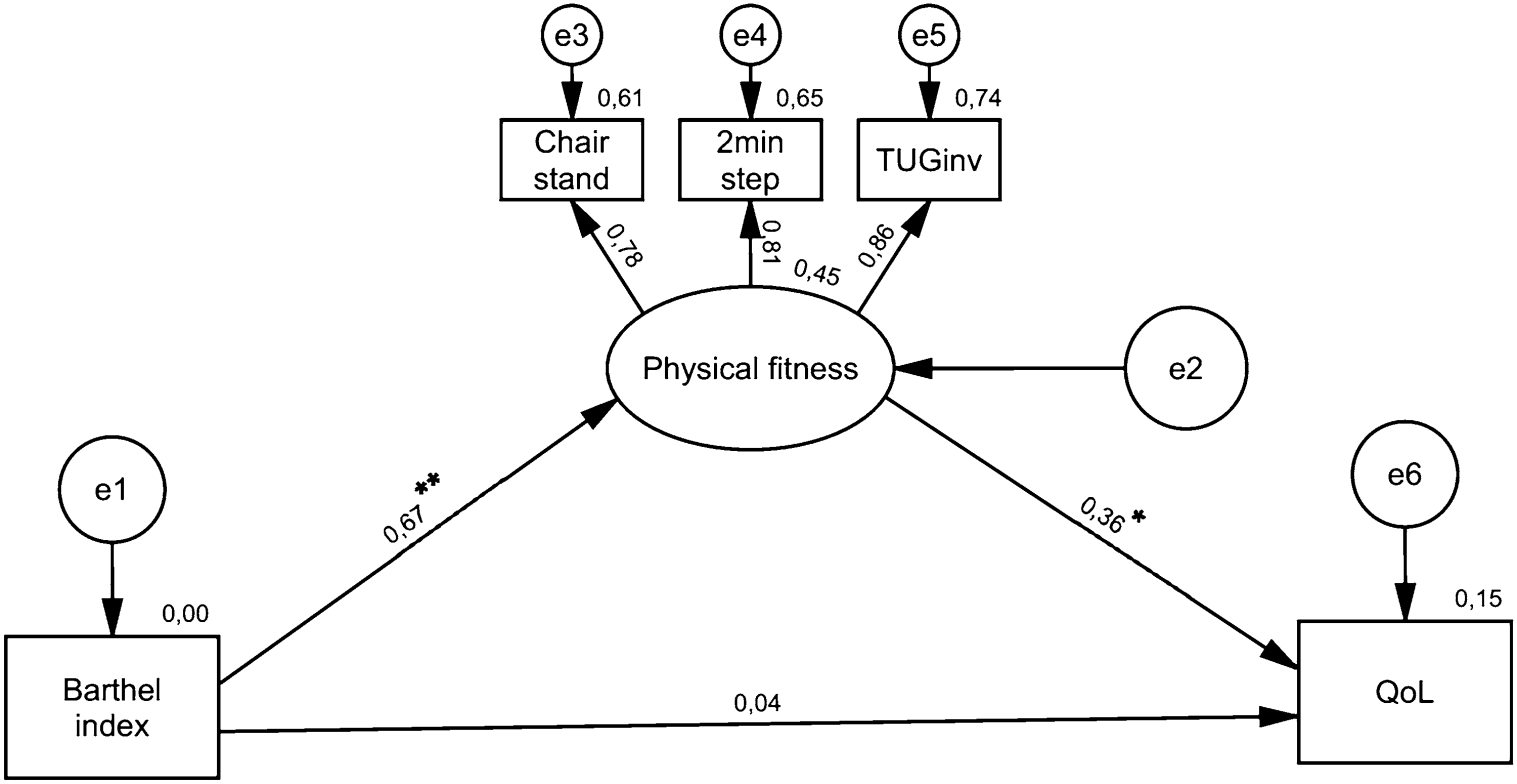
The mediating effect of physical fitness in the relationship between basic ADL and QoL

As measured by the BI, independence in basic ADL had a positive significant direct effect on physical fitness (β = 0.672, p = 0.004), while this exerted a direct effect on QoL (β = 0.360, p = 0.011) (Fig. 1). Independence in basic ADL positively affected QoL mediated by physical fitness (β = 0.242, p = 0.011). No direct effect was observed between independence in basic ADL and QoL (β = 0.043, p = 0.733) (Fig. 2).

The fit of the model was met according to the following parameters: X^2^(4) = 0.124, p = 0.998, SRMR = 0.0037, RMSEA < 0.001, GFI = 1.000, NFI = 0.999 and CFI = 1.000. Based on R^2^ values, the final model accounted for 45% of the variance in physical fitness and 15% in QoL.

## Discussion

Independence in basic ADL was significantly associated with QoL and this relationship seems to be mediated by physical fitness; the same, however, was not observed for cognitive function. Our results demonstrated an absence of statistically significant results when testing cognitive function as a mediator between BI and QoL. This could be somewhat potentially explained by behavioral and psychological symptoms commonly observed across the dementia spectrum rather than by cognitive function per se. Indeed, apathy, aberrant motor behavior and appetite disturbances are associated with higher rate of basic ADL impairments [50]. Also, Banerjee et al. [8] found that behavioral and psychological symptoms in dementia were associated with QoL, whereas cognitive function was not. In accordance with our findings, Clemmensen et al. [24] found no correlation between cognitive function and basic ADLs. Some studies even suggest that rather than global cognitive capacity, some specific cognitive functions (i.e., visual constructive or executive functions) seem to affect the performance of ADL [51]. Also, another study examining the relationship between cognitive function, demographic and disease variables on ADL functionality in individuals with mild to moderate Alzheimer’s disease showed that non-cognitive variables explained 18% of variance. However, the model explained 39% of the variance by adding neuropsychological factors [52]. The authors pointed out that constructional cognitive abilities, figural and verbal memory, longer disease duration and depression were significant predictors of declines in ADL functionality [52]. Longitudinal data demonstrated that dementia severity predicted the decline in basic ADL functioning over time [22, 53], and Vellas et al. [54] also confirmed that a severe impairment in ADAS-cog (e.g., an increase of 7 points or more) was associated with a decline in basic ADLs in a long-term follow-up (18 months). In this sense, it is crucial to further understand whether the deterioration in basic ADL is only driven by physical function or cognitive decline, as basic ADL dependency tends to occur in more severe stages of dementia [27].

As for the mediation role of physical fitness in the relationship between basic ADL independence and QoL, the evidence on cross-sectional and longitudinal studies is consistent, suggesting that specific components of physical fitness such as lower extremity muscle strength and balance are crucial for older adults to maintain a standing posture and independently perform their basic ADL. Indeed, low scores of performance tests such as TUG or chair stand test are associated with future worsening of basic ADL in older adults [55, 56]. The relationship between physical fitness and QoL is less consistent as only a few cross-sectional studies found significant associations between strength or mobility and QoL in older adults [57, 58], whereas to our knowledge no longitudinal study has analyzed this association.

Boyle et al. [59] study suggested that motor performance accounted for a high proportion of the variance in basic ADLs in people with dementia, whereas cognitive function was not significantly associated. These findings support a new evidence that found a correlation between mobility-related functional parameters and ADL performance in people with dementia [60]. Mobility is an important predictor of changes in the QoL of older adults [61] and tends to get impaired as dementia progresses, affecting everyday function and QoL [62]. TUG performance (a marker of functional mobility) is influenced by lower limb strength and balance [63], reinforcing the potential indirect role of physical fitness in QoL parameters. Specific physical fitness components such as strength, flexibility, agility/dynamic balance, and aerobic endurance are related to cognitive function, basic ADL and QoL in people with dementia [12]. Also, sarcopenia-related factors such as lower-extremity function and skeletal muscle mass seem to be associated with QoL [29, 64]. In fact, sarcopenia is a risk factor for poor QoL in older adults and is associated with an increased risk of cognitive impairment [65, 66]. A recent longitudinal study comprising older adults found that poor muscle function, but not reduced lean muscle mass, is the driver of the association between sarcopenia with the incident of dementia and mild cognitive impairment [67]. In this sense, individuals’ functional parameters should be considered when analyzing the QoL of people with dementia.

Although there is clear evidence suggesting that higher independence in basic ADL is associated with better QoL in people with dementia [68], some methodological details seem to blur this relationship. This can be attributed to several factors, of which we highlight the challenge in assessing ADLs or QoL in people with dementia, and the complexity around basic ADL deterioration across the dementia spectrum. Measuring the functional ability of people with dementia is challenging as self-reported measures in this population may raise concerns over reliability [69]. Frequently, proxy/carer/informant reports or performance measures are used because they seem to be more reliable in this population, but assessment instruments vary greatly across studies due to the lack of a standard measure [69]. We used BI to assess independence on basic ADL. BI is a generic instrument that presents acceptable psychometric proprieties in the geriatric population [70, 71]. However, several reports assume that cognitive abilities influence its scores [51]. Recent evidence even suggests that BI is not appropriate to assess ADL independence in people with dementia directly [72]. To address this issue, our data was obtained by proxy report. In what concerns the second highlighted factor, it is known that ADL performance deteriorates differently for every basic activity and that it is dependent on the disease severity [73]. There may be a hierarchy in the functional decline of ADLs, since participants tend to lose the ability to bathe independently before losing other skills, such as dressing, using the toilet, and transferring [74]. Also, the ability to eat autonomously seems well-preserved as cognition declines [27]. This hierarchy framework is supported by well-known longitudinal data showing that older adults tend to lose the ability to perform activities that require lower extremity strength earlier than upper extremity strength [75]. Basic ADL performance has a different effect on QoL across dementia stages [76], possibly due to the carers’ perception of the impact of basic ADL dependency on QoL and their lives, particularly in severe stages [73].

In our work, we outlined the importance of physical fitness, particularly lower extremity tests, when examining the relationship between basic ADL independence and QoL. However, further longitudinal studies with large samples size are also needed to explore the potential bidirectional relationship between cognitive function, disability in basic ADL and QoL. Determining other potential mediators of these relationships such as behavioral and psychological symptoms is also to be considered.

Some limitations must be acknowledged in this study, and the results should thus be interpreted with caution. Primarily, our results might not be generalizable due to our small sample size, the sample’s specific characteristics (i.e., high levels of independence in basic ADL, unequal distribution of participants from both genders and dementia subtypes), and lack of data on disease severity. In addition, both independence on ADL and QoL questionnaires relied on carers’ reports, and evidence suggests an apparent discrepancy between self-reports and caregiver reports [27], possibly induced by carer’s burden; therefore, we cannot exclude potential bias.

## Conclusion

Our study findings present contributions to the available knowledge about the mediating effect of physical fitness (i.e., lower body function, aerobic capacity, and mobility) on the relation between basic ADL independence and QoL of older adults with dementia, reinforcing the need to address, measure and control physical capacities, and not only direct dementia-related factors like cognitive function, neuropsychiatric symptoms, and disease duration. Future longitudinal studies would be important to explore the temporal relationship between physical and cognitive function and its contribution to basic ADL dependency and QoL in individuals with dementia.

## Data Availability

The datasets used and/or analyzed during the current study are available from the corresponding author upon reasonable request.
